# Ipilimumab and Nivolumab-Induced Colitis in a Patient With Recurrent Metastatic Melanoma

**DOI:** 10.7759/cureus.14414

**Published:** 2021-04-11

**Authors:** Hamid-Reza Moein, Brian Rutledge, Rafic Beydoun, Murray N Ehrinpreis

**Affiliations:** 1 Internal Medicine, Sinai-Grace Hospital/Detroit Medical Center, Wayne State University School of Medicine, Detroit, USA; 2 Gastroenterology, Wayne State University Detroit Medical Center, Detroit, USA; 3 Pathology, Wayne State University Detroit Medical Center, Detroit, USA; 4 Gastroenterology, Wayne State University, Detroit, USA

**Keywords:** immune-mediated colitis, immune checkpoint inhibitor colitis, drug-induced colitis

## Abstract

Ipilimumab and nivolumab are immune checkpoint inhibitors that have recently been used in the treatment of metastatic melanoma and other cancers. Immune-mediated colitis is one of their adverse events that need to be differentiated from low-grade diarrhea as one of the most common side effects. A 51-year-old woman with relapsed metastatic melanoma presented with intractable diarrhea, nausea, vomiting, and generalized abdominal pain. The patient had been treated with ipilimumab and nivolumab in the past two months. The infectious workup was inconclusive. Colonoscopy demonstrated severe colitis, and biopsies were consistent with colitis. Combination chemotherapy was stopped. The patient was treated with intravenous and oral steroids, and her symptoms improved. A combination of ipilimumab and nivolumab increases the chance of immune-mediated colitis, and steroids should be started promptly to avoid complications such as bowel perforation and toxic megacolon.

## Introduction

Drug-induced colitis is historically found as a reaction to nonsteroidal anti-inflammatory and antibiotic medications [1].^ ^After the advent of immune checkpoint inhibitors for the treatment of metastatic melanomas and other cancers, immune-mediated colitis was reported as one of the immune-related adverse events (irAEs) [2]. Ipilimumab and nivolumab are monoclonal antibodies against cytotoxic T-lymphocyte-associated protein 4 (CTLA-4) and programmed cell death protein 1 (PD-1), respectively [1,3-4]. They both increase the activity of T cells against cancerous cells and can simultaneously result in the stimulation of T cells against non-cancerous tissues such as the gastrointestinal (GI) tract [3]. The reported incidence of colitis is about five times greater with ipilimumab as compared to nivolumab and is 13.6% with combination therapy [3].

This article was partly presented as a virtual meeting abstract at the 2020 American College of Gastroenterology.

## Case presentation

**Figure 1:**
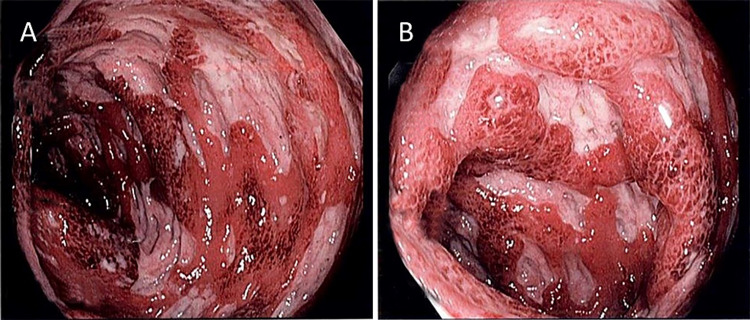
Left-sided colon micrographs obtained by colonoscopy A and B: Demonstrating friable, scattered, punched-out ulcers and inflammation/colitis in the colon.

**Figure 2:**
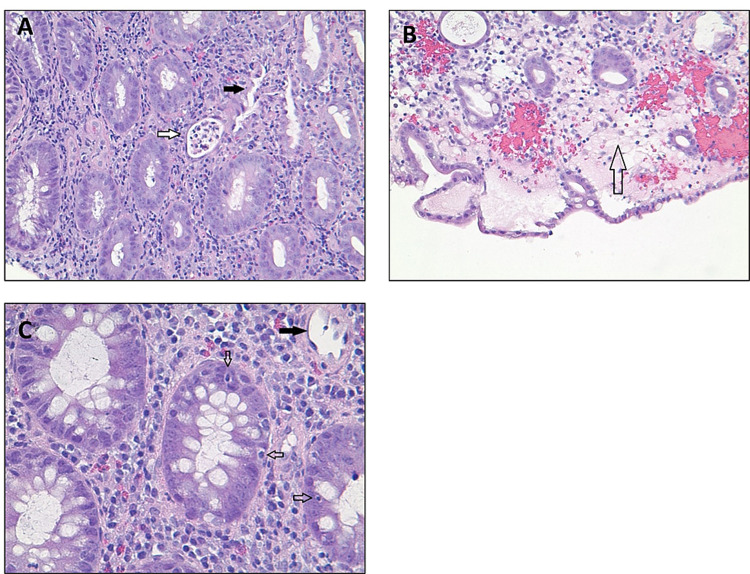
Histologic images from colon biopsy A: x200 magnification; hematoxylin and eosin staining, demonstrating inflammatory cells in the lamina propria, crypt abscess (hollow arrow), and crypts destruction and dropout (solid arrow). B: x200 magnification; surface epithelial injury with edema (hollow arrow). C: x400 magnification; apoptotic bodies (hollow arrow) and crypts dropout (solid arrow).

A 51-year-old Caucasian woman with relapsed, metastatic, BRAF gene mutation-positive melanoma presented with intractable nausea, vomiting, abdominal pain, and copious diarrhea. Diarrhea began about four weeks prior to presentation following the second cycle of ipilimumab and nivolumab. After the third cycle, diarrhea worsened with more than seven episodes of non-bloody diarrhea per day, unresponsive to loperamide and diphenoxylate. Diarrhea was associated with moderate cramping and generalized abdominal pain with mild alleviation after bowel movement. The patient also had nausea and multiple episodes of vomiting (non-bilious and non-bloody) and reported a recent 8 lbs weight loss. Physical exam was benign, without abdominal tenderness. On admission, serum sodium and potassium were 132 mMol/L and 2.3 mMol/L, respectively. She underwent aggressive fluid and electrolyte replacement. Stool culture and Clostridioides difficile deoxyribonucleic acid (DNA) amplification stool test were negative. Colonoscopy revealed significant, severe colitis in the transverse and descending colon (Figures 1A-1B). The colonoscope was not advanced further for safety reasons. Mild-moderate colitis in the sigmoid colon, with erythema, friable scattered punched-out ulcers, and proctitis, was also observed (Figures 1A-1B). Multiple colon biopsies were obtained, which showed mild architectural abnormality with ulceration, focal crypt abscess, apoptotic bodies, and inflammatory cells in lamina propria (Figures 2A-2C). Cytomegalovirus DNA was negative. No granuloma or dysplasia was observed histologically. Immune checkpoint inhibitors were discontinued. Intravenous methylprednisolone (1 mg/kg dose) was initiated for three days with a response in 24 hours by decreasing diarrhea. The patient was discharged with 100 mg prednisone daily and her symptoms subsequently improved. Steroids were tapered over four months, and she did not have any recurrence of colitis after the one-year follow-up.

## Discussion

Immune-mediated colitis is one of the side effects of immune checkpoint inhibitors, which are recently being used more frequently for the treatment of several cancers, including metastatic melanoma [1,3-4]. The majority of immune checkpoint-induced diarrheas are mild (grades 1 and 2) and grades 3-5 are rare [1,3]. The incidence of ipilimumab-induced colitis is higher than nivolumab (5.7%-9.1% vs. 0.7%-1.6%) [3]. Combination therapy increases the frequency of colitis in these patients with an incidence of 13.6% [3]. Diarrhea, abdominal pain, hematochezia, fever, and vomiting constitute the most common to least common symptoms in patients with immune checkpoint inhibitor colitis [3]. Ipilimumab (but not nivolumab) causes dose-dependent colitis [3]. The median onset of symptoms after the first infusion of ipilimumab is about four weeks and for nivolumab, this varies from two to four months to up to two years [3]. The number of reported nivolumab-induced colitis cases in the literature is limited. Cañete et al. reported only 19 cases published till 2019 [4]. Cheung et al. reported 41 cases of nivolumab-induced colitis and 51 cases of immune colitis after combined immune checkpoint inhibitor therapy [5].

The exact pathophysiology of immune-mediated colitis is unknown, however, the hyperproliferation and hyperactivation of T cells and lymphocyte infiltration are proposed mechanisms [6-7]. Coutzac et al. demonstrated distinct inflammatory infiltrates and the tumor necrosis factor-alpha (TNF-alpha) secretion property in histology samples obtained from CTLA-4 or PD-1-induced colitis [7]. A detailed history and complete laboratory workup to rule out other causes of diarrhea and colitis (such as Clostridioides difficile infection, cytomegalovirus infection, hyperthyroidism, and celiac disease) is essential in the diagnosis of immune-mediated colitis [3]. Clinical symptoms do not always correlate with mucosal colitis, therefore, timely colonoscopy is crucial for these patients [3,8]. Abu-Sbieh et al. demonstrated that colonoscopy within seven days decreased symptoms and steroid therapy duration in patients with immune-mediated colitis [8]. Colonoscopy findings range in severity from normal (37%) to pancolitis (23%-40%) with mucosal ulcerations (27%-40%) [3,7]. The frequency of these patterns are slightly different between ipilimumab and nivolumab, with the latter presenting more commonly with pancolitis [7]. Acute infiltration of neutrophils and other immune cells into the lamina propria is the most common histologic finding in these patients [1,7], which was also detected in our patients.

In this case, we presented a patient with metastatic melanoma who developed colitis after five to six weeks from the first infusion of combined ipilimumab and nivolumab. Whether ipilimumab, nivolumab, or both were the culprits in causing immune-mediated colitis is not clear, as both present with similar clinical and histologic findings. The fact that symptoms started about one month from the infusion favors ipilimumab-induced colitis. On the other hand, the extensive colitis pattern in our patient favors nivolumab-induced colitis. 

Steroids are the first line of therapy for immune-mediated colitis [3-4]. However, in 12.5% to 25% of patients, steroids fail [3-4]. In these cases, anti-TNF alpha (infliximab) [3-4]​​​​​​, vedolizumab (an a4b7 antagonist) [3], and fecal transplantation have been utilized for the treatment of steroid-refractory cases [9]. Histologic severity has been reported as an indicator of infliximab requirement for treatment (the more severe less likely to respond to steroids only) [3]. More recently, ustekinumab (interleukin-12/23 receptors blockers) [10] and tofacitinib (Janus Kinas inhibitor) [11] were reported in the treatment of refractory cases.

Intestinal superinfection with Clostridioides difficile or cytomegalovirus should be ruled out before starting any immunosuppressive therapy [4]. A detailed approach to patients with immune-mediated colitis is illustrated by Bellaguarda and Hanauer [3]. Our patient responded well to steroid therapy. Factors that determine response to steroid therapy for some patients versus others are not studied widely and further research is warranted.

## Conclusions

Cancer patients under treatment with immune checkpoint inhibitors, especially combined therapy, who present with worsening symptoms of colitis and diarrhea should be evaluated promptly. A colonoscopy is indicated after negative infectious workup in those who do not respond to conservative treatment. Once the diagnosis of immune-mediated colitis is reached, conservative treatment with steroid therapy should be started. Patients should be monitored closely to avoid complications such as bowel perforation and toxic megacolon.
